# A scoring model based on clinical factors to predict postoperative moderate to severe acute respiratory distress syndrome in Stanford type A aortic dissection

**DOI:** 10.1186/s12890-023-02736-6

**Published:** 2023-12-21

**Authors:** Maozhou Wang, Songhao Jia, Xin Pu, Lizhong Sun, Yuyong Liu, Ming Gong, Hongjia Zhang

**Affiliations:** 1 Department of Cardiac Surgery, Beijing Anzhen Hospital, Capital Medical University, Beijing, China; 2 Department of Interventional Therapy, Beijing Anzhen Hospital, Capital Medical University, Beijing, China

**Keywords:** Machine learning, Prediction model, Acute respiratory distress syndrome, Stanford type A aortic dissection

## Abstract

**Background:**

Postoperative acute respiratory distress syndrome (ARDS) after type A aortic dissection is common and has high mortality. However, it is not clear which patients are at high risk of ARDS and an early prediction model is deficient.

**Methods:**

From May 2015 to December 2017, 594 acute Stanford type A aortic dissection (ATAAD) patients who underwent aortic surgery in Anzhen Hospital were enrolled in our study. We compared the early survival of MS-ARDS within 24 h by Kaplan–Meier curves and log-rank tests. The data were divided into a training set and a test set at a ratio of 7:3. We established two prediction models and tested their efficiency.

**Results:**

The oxygenation index decreased significantly immediately and 24 h after TAAD surgery. A total of 363 patients (61.1%) suffered from moderate and severe hypoxemia within 4 h, and 243 patients (40.9%) suffered from MS-ARDS within 24 h after surgery. Patients with MS-ARDS had higher 30-day mortality than others (log-rank test: p-value <0.001). There were 30 variables associated with MS-ARDS after surgery. The XGboost model consisted of 30 variables. The logistic regression model (LRM) consisted of 11 variables. The mean accuracy of the XGBoost model was 70.7%, and that of the LRM was 80.0%. The AUCs of XGBoost and LRM were 0.764 and 0.797, respectively.

**Conclusion:**

Postoperative MS-ARDS significantly increased early mortality after TAAD surgery. The LRM model has higher accuracy, and the XGBoost model has higher specificity.

**Supplementary Information:**

The online version contains supplementary material available at 10.1186/s12890-023-02736-6.

## Introduction

ARDS is a common complication in Stanford type A aortic dissection (TAAD), with an incidence of 10–50% [1, 2]. ARDS significantly increases the length of hospitalization and mortality [3, 4]. However, the specific mechanism underlying the high incidence of ARDS after TAAD is still unclear. The early prediction of high-risk patients with ARDS after surgery would help optimize the management of ventilators and in exploring the underlying disease mechanisms [5].

There are many factors affecting the occurrence and prognosis of postoperative ARDS [6, 7]. However, TAAD may have some specific high-risk factors, such as involvement of the bronchial artery by aortic dissection or hypothermic circulatory arrest during surgery [8]. For these patients, special respiratory management may be required. According to the 2017 clinical practice guidelines of the American Thoracic Society/European Society of Intensive Care Medicine/Society of Intensive Care Medicine, high PEEP is beneficial for patients with moderate to severe ARDS (MS-ARDS) but not for mild ARDS patients [9]. Prone ventilation for more than 12 h per day is recommended for severe ARDS patients [10]. However, patients undergoing cardiac surgery cannot perform prone ventilation for a long time due to sternal problems and circulation instability [11]. In addition, patients with MS-ARDS may need further respiratory management, such as extracorporeal CO2 removal [12, 13]. The treatment and management of MS-ARDS are different from those of mild ARDS. Early detection of high-risk patients with MS-ARDS and the identification of some high-risk factors for MS-ARDS would be beneficial for the implementation of preventive measures before MS-ARDS.

This study aims to identify the clinical factors associated with postoperative moderate to severe ARDS in STAAD patients and develop predictive models for early detection. We present the content of the article according to the STROBE Checklist.

## Materials and methods

### Patients

From May 2015 to December 2017, 597 TAAD patients received surgery at Beijing Anzhen Hospital. The inclusion criteria were TAAD patients who received aortic surgery and were ≥ 18 years old. The exclusion criteria were death during surgery or death within one day after surgery. There was one patient aged < 18 years old, and 2 patients died within one day after surgery. Finally, a total of 594 patients were enrolled in our study. The Anzhen Hospital Ethics Committee approved the protocol of this retrospective study in April 2018 (No. 2,018,004) and waived the need for informed consent from each patient.

### Definitions and endpoints

Aortic dissection is divided into 1–3 types and simple or complex types according to the Sun’s classification [14]. Hypoxemia is defined as OI ≤ 300 mmHg and moderate to severe hypoxemia is defined as OI ≤ 200 mmHg. According to the Berlin definition [15], ARDS is defined as OI ≤ 300 mmHg with chest X-ray showing pulmonary infiltration and PEEP ≥ 5 cmH2O without clear reasons (such as left heart failure or pneumonia) within 24 h postoperatively. MS-ARDS was defined as OI ≤ 200 mmHg with ARDS. The main endpoint is 30-day all-cause death. The secondary endpoint was moderate to severe hypoxemia and MS-ARDS within 24 h after surgery.

### Data processing and statistics

Variables missing more than 10% of the values were excluded; for variables missing less than 10% of the values, the data were interpolated (5 times interpolation). We divided the patients into two groups according to whether they had moderate to severe hypoxemia within 4 h postoperatively: patients with moderate to severe hypoxemia and patients without moderate to severe hypoxemia. We also divided the patients into two groups based on whether they experienced MS-ARDS within 24 h postoperatively: patients without MS-ARDS and patients with MS-ARDS. Kaplan–Meier curves and log-rank tests were used to compare the 30-day survival rate between the two groups. Independent t-tests were used for continuous variables conforming to a normal distribution, and Mann–Whitney U tests were used for continuous variables without a normal distribution. The χ2 chi-square test was used for categorical variables. Bilateral *P* < 0.05 was considered statistically significant. IBM SPSS 26 was used for all of the above statistics. Prism was used to draw the Kaplan–Meier curve.

### Model construction

We first performed univariable logistic analysis and excluded variables with *P* > 0.1 from the analysis. We used a total of 30 variables to construct the models. XGBoost and logistic regression algorithms were used to construct the models. Accuracy and AUC were used to evaluate model effectiveness. RStudio version 4.1.1 was used for model construction. We used the following R packages: XGBoost, rms, nomogram, pROC, and Matrix.

## Results

### Baseline characteristics

We compared the baseline characteristics between the two groups (non-MS-ARDS vs. MS-ARDS), as shown in Table 1. MS-ARDS patients were older (50 years old vs. 46 years old; *p* < 0.001) and had a higher BMI (26.37 kg/m2 vs. 25.39 kg/m2, *p* < 0.001)), and more patients in this group suffered from hypertension preoperatively (62.6% vs. 49.1%, *p* < 0.001). There was no significant difference in the incidence of patients with descending aortic dissection between the two groups (71.5% vs. 75.3%, *p* = 0.347). The differences in postoperative variables are shown in Table 2. The operation time, cardiopulmonary bypass time, aortic occlusion time, and cardiac arrest time were higher in MS-ARDS patients. A comparison of the missing variables between the two groups is presented in Supplemental Table 1.

**Table 1:** Baseline of MS-ARDS after TAAD surgery^a^

Variable	All patients	Non-MS-ARDS	MS-ARDS	*p*-value
	*n* = 594	*n* = 351 (59.1)	*n* = 243 (40.9)	
Age (years), median (IQR)	49(16)	46(16)	51(14)	< 0.001**
Male, n (%)	437(73.6)	264(75.2)	173(71.2)	0.298
BMI (kg/m2)^b^, median (IQR)	25.95(4.12)	25.35(4.69)	26.89(4.64)	< 0.001**
Hypertension, n (%)	333(56.1)	158(45.0)	175(72.0)	< 0.001**
Diabetes, n (%)	20(3.7)	12(3.4)	8(3.3)	0.104
Coronary artery disease, n (%)	9(1.5)	6(1.7)	3(1.2)	0.170
Smoking, n (%)	208(35.0)	118(33.6)	90(37.0)	0.431
D-dimer (ng/ml), median (IQR)	2043.5(1912)	1902(1884)	2203(2148)	0.018*
COPD^c^, n (%)	3(0.5)	3(0.9)	0(0)	0.274
Descending aortic dissection, n (%)	434(73.1)	251(71.5)	183(75.3)	0.347

^a^Moderate to severe acute respiratory distress syndrome; *STAAD *Stanford type A aortic dissection. ^b^*BMI *Body mass index. ^c ^Chronic obstructive pulmonary disease.**:*p *< 0.01;*:*p < *0.05.

**Table 2:** Perioperative factors between MS-ARDS and non-MS-ARDS undergoing STAAD surgerya

Variable	All patients	Non-MS-ARDS	MS-ARDS	*p-value*
	*n*=594	*n*=351 (59.1)	*n*=243 (40.9)	
Operation time (hours), median (IQR)	7.8 (2.1)	7.5 (1.8)	8.0 (2.3)	< 0.001**
Cardiopulmonary time (min), median (IQR)	205 (57)	198 (58)	217 (61)	< 0.001**
Aortic occlusion time (min)b, median (IQR)	114 (42)	110 (42)	119 (45)	0.002**
Cardiac arrest time, median (IQR)	22 (10)	21 (10)	22 (12)	0.005**
Proximal aorta management				
Ascending aorta replacement, n (%)	131 (22.1)	64 (18.2)	67 (27.6)	0.009**
Bentall, n (%)	249 (41.9)	172 (49.0)	77 (31.7)	< 0.001**
Wheat, n (%)	2 (0.3)	1 (0.3)	1 (0.4)	1
David, n (%)	1 (0.2)	0 (0)	1 (0.4)	1
Aortic arch management				
Partial arch replacement, n (%)	30 (5.1)	17 (4.8)	13 (5.3)	0.850
Total arch replacement, n (%)	434 (73.1)	251 (71.5)	183 (75.3)	0.347
Concomitant CABG^b^, n (%)	34(5.7)	13(3.7)	21(8.6)	0.018*
Concomitant mitral valve replacement, n (%)	8(1.3)	6(1.7)	2(0.8)	0.482
Concomitant tricuspid valvuloplasty, n (%)	3(0.5)	3(0.9)	0(0)	0.274
ASA anesthesia score, n (%)	360(60.6)	204(58.1)	156(64.2)	0.925
1, n (%)		1(0.3)	1(0.4)	
2, n (%)		6()	3	
3, n (%)		86	72	
4, n (%)		98	71	
5, n (%)		13	9	
The nasopharyngeal temperature during circulatory arrest	23.9(1.7)	23.8(1.7)	24.0(1.6)	0.326
The anal temperature during circulatory arrest	25.3(1.8)	25.2(2.1)	25.4(1.6)	0.088

^a^*MS-AHRF* Moderate to severe acute respiratory distress syndrome, *STAAD* Stanford type A aortic dissection, ^b^ Coronary artery bypass grafting. **:*p *< 0.01, *:*p < *0.05

### Early outcomes

**Fig. 1:**
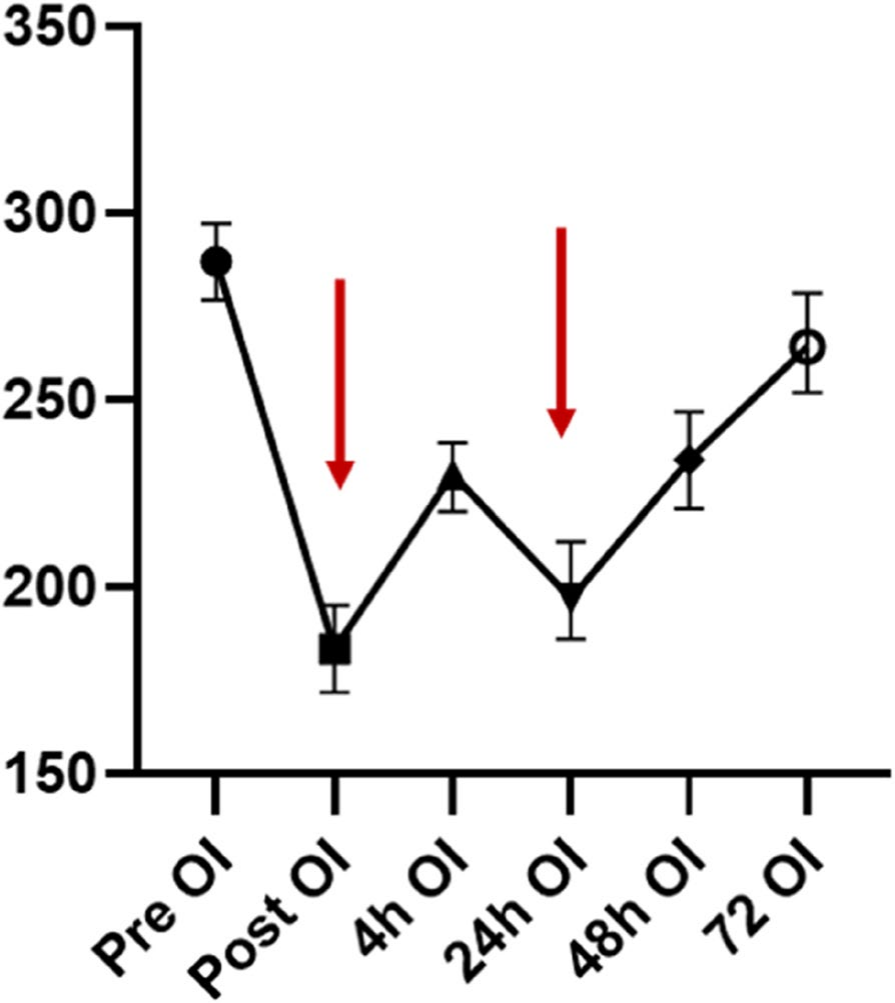
OI of all patients at different time points

**Fig. 2:**
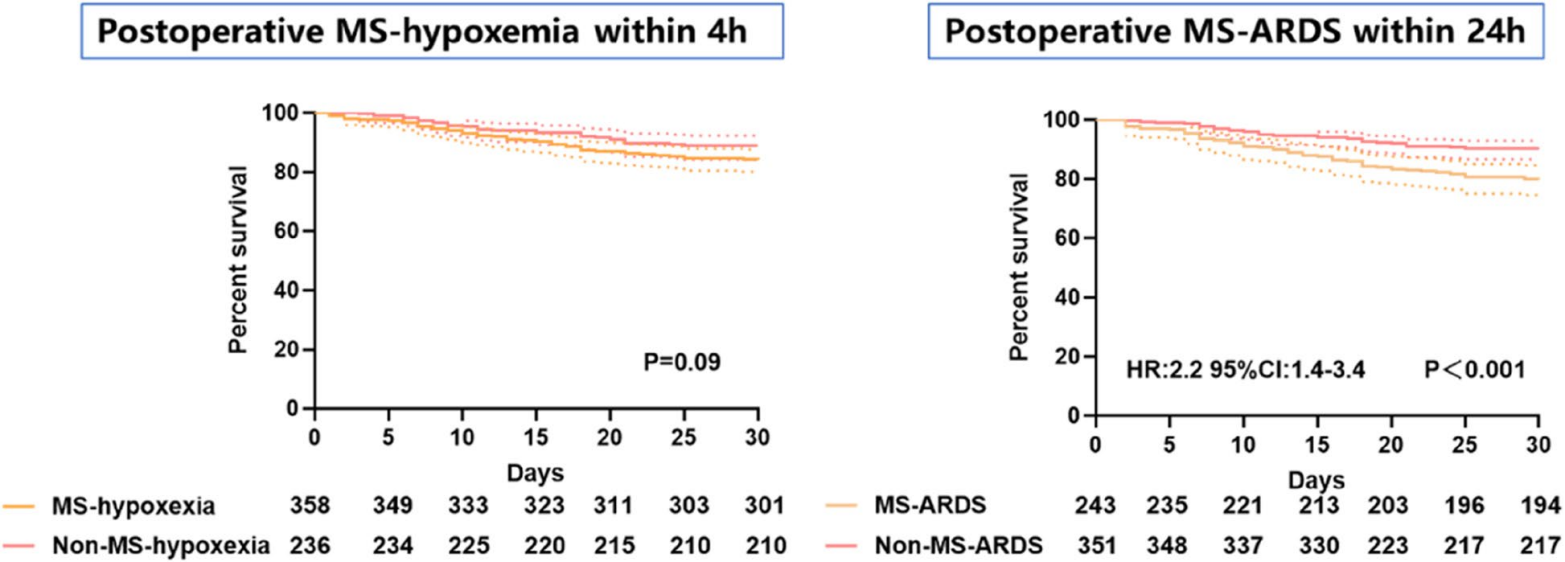
The early mortality rate of patients; **a **Early mortality of patients with or without early hypoxemia within 4 h of surgery; **b** Early mortality of patients with or without early hypoxemia within 24 h of surgery

As shown in Fig. 1, we analyzed the OI of all patients at different time points. We identified two main time points at which the OI decreased in patients with STAAD: immediately after surgery and 24 h after surgery. In addition, we analyzed the early survival of early hypoxemia and 24-hour MS-ARDS patients, and we found that there was no significant difference in the early mortality of patients with or without early hypoxemia within 4 h of surgery (*P* = 0.09, Fig. 2a). However, at 24 h, the development of MS-ARDS significantly increased early mortality (HR: 2.2 95% CI: 1.4–3.4, *P* < 0.001, Fig. 2b). Therefore, we explored and established models of the risk factors for 24-hour postoperative MS-ARDS.

### Model results

**Fig. 3:**
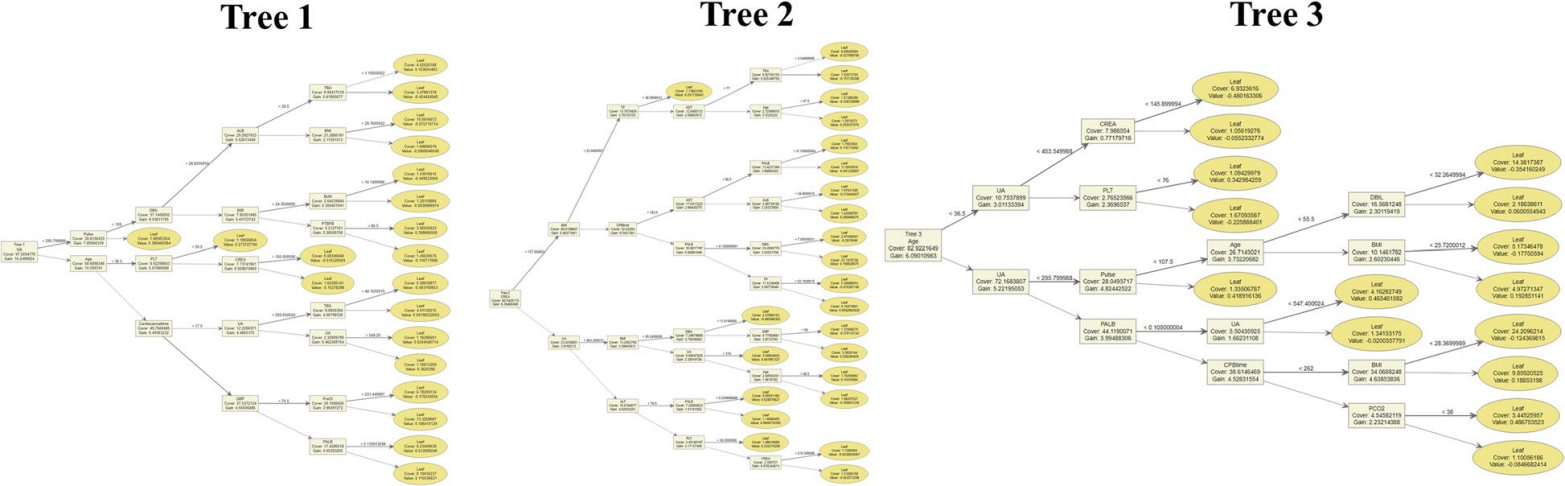
The first three trees of XGBoost

**Fig. 4:**
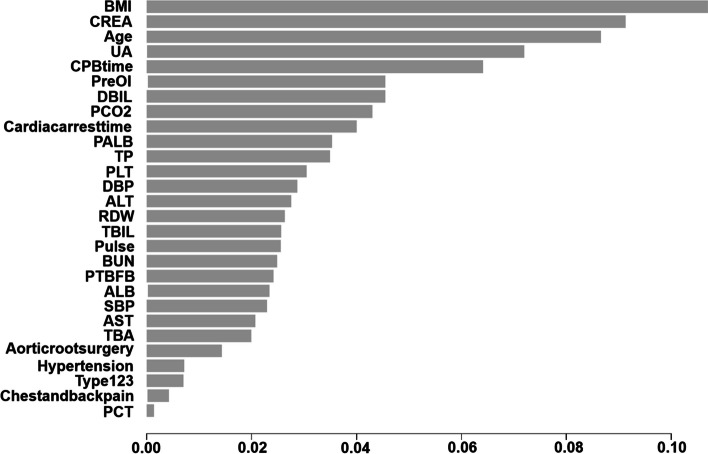
The 10 most important variables affecting MS-ARDS

**Fig. 5:**
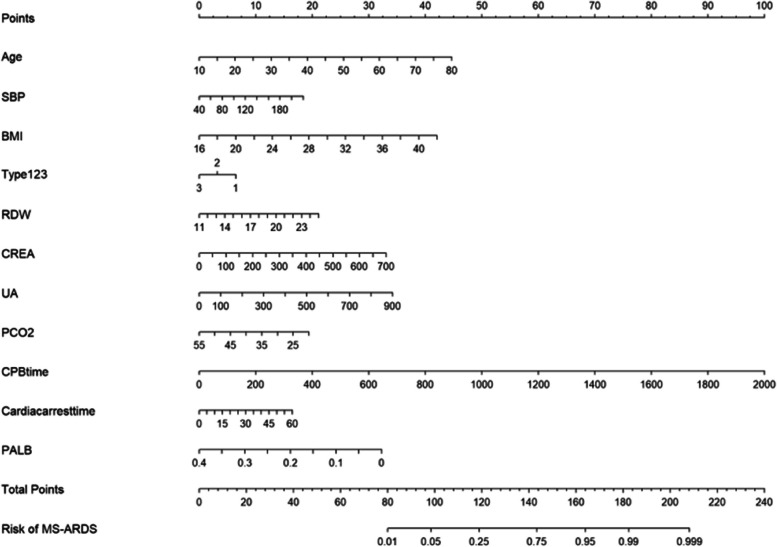
The nomogram of the model

There were 116 variables in total, of which the number of missing values of 30 variables was greater than 10% (Supplemental Table 2), and we excluded them. We conducted univariate logistic analysis on the remaining 86 variables (Supplemental Table 3) We identified 30 variables related to 24-hour MS-ARDS in the univariate logistic analysis. These variables are shown in Table 3 (other variables with *P* > 0.1 are shown in Supplemental Table 2). As shown in Table 3, we conducted heterogeneity tests on the interaction terms between cardiopulmonary bypass time and cardiac arrest time, and the results showed that there was no interaction on MS-ARDS between the two factors (*P* = 0.412). In addition, we also conducted heterogeneity tests on the interaction terms between Concomitant CABG and Bentall surgery, and found that there was also no interaction on MS-ARDS between the two factors (*P *= 0.853). As shown in Supplemental Fig. 1, we discretized the continuous variables in this section and reconstructed a forest map of their impact on the postoperative MS-ARDS. According to a 7:3 ratio, 415 patients were randomly assigned to the training set, and 179 patients were randomly assigned to the test set. We established a logistic regression model and XGBoost model with these 30 variables using the training set. XGBoost generated a total of 29 trees, and the first three trees are shown in Fig. 3. In addition, we analyzed which variables were most important in the XGBoost model. The 10 most important variables affecting MS-ARDS are body mass index, creatinine, age, uric acid, cardiopulmonary bypass time, preoperative OI, direct bilirubin, preoperative PCO2, cardiac arrest time, and albumin (Fig. 4). The max-depth of the tree is three, and the eda is six. The bidirectional stepwise optimization method was used for logistic regression, and 11 variables (age, systolic blood pressure, body mass index, Sun’s classification, red blood cell distribution width, creatinine, uric acid, PCO2, CPB time, cardiac arrest time and albumin) were included in the final logistic regression model. The nomogram is presented in Fig. 5. The discretized nomogram can be found in supplemental Fig. 2. We use Sun’s classification as the basis for stratified sampling, with 3/5 of the data as the training set, and 2/5 of the data as the test set. Finally, we found that the AUC of the logistic regression model test set is 0.71, and the AUC of XGboost is 0.59 (Supplemental Fig. 3). It also indicates that the logistic model has good predictive performance under different patient feature stratification of aortic dissection.

**Table 3:** Univariate logistic regression of MS-ARDSa within 24 h (variables with *P*<0.1)

Variables	OR	95%CI	*P-value*
Self-related factors			
Age	1.042	1.025–1.059	< 0.001
Pulse	1.013	1.001–1.025	0.033
Systolic blood pressure	1.008	1.000-1.017	0.061
Diastolic blood pressure	1.017	1.005–1.029	0.004
BMI^b^	1.164	1.109–1.223	< 0.001
Hypertension	1.869	1.335–2.617	< 0.001
Diabetes	2.227	0.897–5.533	0.085
Inflammatory factors			
PLT^c^	0.995	0.992–0.998	< 0.001
PCT^d^	0.006	0.000-0.008	< 0.001
BUN^e^	1.126	1.076–1.179	< 0.001
CREA^f^	1.011	1.007–1.014	< 0.001
UA^g^	1.005	1.003–1.006	< 0.001
ALT^h^	1.001	1.000-1.002	0.08
AST^i^	1.001	1.000-1.001	0.049
TBA^j^	1.106	0.996–1.228	0.06
PTBFB^k^	0.991	0.984–0.998	0.016
RDW^l^	1.166	1.037–1.311	0.01
TBIL^m^	1.006	1.000-1.011	0.062
DBIL^n^	1.011	1.002–1.021	0.018
Surgical factors			
Sun’s classification(Type 2 vs. Type 1)	0.664	0.436–1.010	0.056
Sun’s classification(Type 3 vs. Type 1)	0.356	0.220–0.578	< 0.001
CPB time^o^	1.009	1.005–1.013	< 0.001
Cardiac Arrest time	1.028	1.010–1.047	0.003
Concomitant CABG^p^	2.456	1.205–5.008	0.013
Proximal surgery			
Bentall	0.506	0.346–0.740	< 0.001
pulmonary edema			
TP^q^	0.97	0.952–0.988	0.001
ALB^r^	0.945	0.916–0.974	< 0.001
PALB^s^	0.004	0.000-0.190	0.005
Preoperative lung injury factors			
PCO2^t^	0.956	0.924–0.989	0.009
PreOI^u^	0.998	0.997-1.000	0.098
Preoperative aspiration			
Chest and back pain	1.383	0.994–1.924	0.054

^a^Moderate to severe acute respiratory distress syndrome, ^b^Body mass index, ^c^Platelet, ^d^Procalcitonin, ^e^Blood urine nitrogen, ^f^Creatinine, ^g^Uric acid, ^h^Alanine aminotransferase, ^i^Aspartate transaminase, ^j^Total bile acid, ^k^Plasma prothrombin time, ^l^Red blood cell distribution width, ^m^Total bilirubin, ^n^Direct bilirubin, ^o^Cardiopulmonary bypass time, ^p^Concomitant coronary bypass grafting surgery, ^q^Total plasma protein, ^r^Albumin; ^s^Prealbumin, ^t^Carbon dioxide partial pressure, ^u^Preoperative oxygenation index

### Comparison of model prediction efficiency

**Fig. 6:**
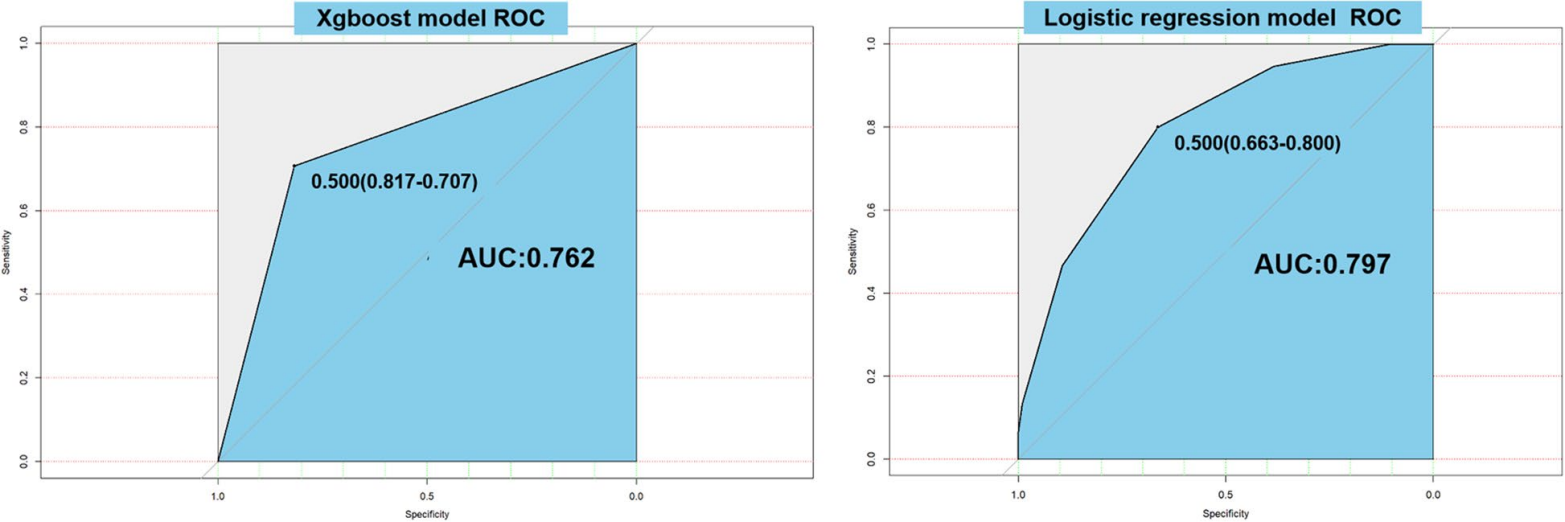
The areas under the curve (AUCs) of the two models; **a** XGBoost model ROC; **b** Logistic regression model ROC

As shown in Fig. 6, the accuracies of the logistic regression model and XGBoost model are 0.800 and 0.707, respectively. The precision is 0.663 and 0.875. The areas under the curve (AUCs) of the two models were 0.79 and 0.76 in the test set.

## Discussion

Determining whether or what measures should be taken to manage ARDS after STAAD surgery has not yet been clarified. We found that postoperative MS-ARDS significantly increased 30-day mortality. Thirty preoperative and perioperative variables were associated with MS-ARDS after surgery. Based on these variables, we established postoperative MS-ARDS risk models, and we clarified the key factors affecting postoperative MS-ARDS. In addition, we used the model to identify high-risk patients with postoperative MS-ARDS that would benefit from early respiratory management.

The reasons for the high incidence of MS-ARDS after type A aortic dissection are multifaceted. First, the operation time and cardiopulmonary bypass time of type A aortic dissection are relatively long, and lung ischemic injury may be more severe than that in regular cardiac surgery. Second, the systemic inflammatory response is common in patients undergoing type A aortic dissection, and this response is closely related to the occurrence of MS-ARDS [16]. Prealbumin and uric acid have also been reported to be related to inflammation [17, 18]. In addition, aortic dissection can involve bronchial arteries, resulting in the involvement of pulmonary nutrient vessels, which can lead to pulmonary ischemia. Most of the bronchial arteries originate from the descending aorta [19]. However, in our study, there was no significant relationship between descending aorta involvement and MS-ARDS, which may be due to the difficulty of distinguishing the degree of bronchial artery involvement. Finally, certain surgical methods, such as hypothermic circulatory arrest, differ from those used for simple cardiopulmonary bypass and may also cause MS-ARDS. At the time of circulatory arrest, the lung is in a state of complete ischemia due to the reduction in the bronchial artery blood supply, which could lead to more serious lung ischemia–reperfusion injury. Our research has found most of the factors that affect postoperative MS-ARDS. But blood product transfusion which has been reported associated with ARDS and its prognosis not included in our models [20]. This may have an impact on the accuracy of our models.

Moderate to severe hypoxemia immediately after surgery may be caused by acute lung ischemia, but it is still unclear why OI decreased at 24 h in some patients, similar to other reports [1]. The improvement in OI at 4 h after surgery indicates that ventilator management is effective over the short term after surgery. Regarding the decrease in OI at 24 h, there may be two reasons why this occurs. On the one hand, there are some factors of continuous injury in the body, which may be due to the influence of the postoperative hypercoagulable state and high levels of inflammatory factors. On the other hand, there may be secondary lung injury after surgery. Most patients need to maintain high-concentration oxygen inhalation after aortic dissection surgery. However, studies have shown that high-concentration oxygen inhalation may expedite the frangibility of pulmonary surfactant, thereby causing atelectasis [21].According to the 2012 Berlin standard [15], the onset of ARDS in most people occurs within 72 h after direct or indirect injury. At the same time, multiple studies and American-European Consensus Conference have shown that the 24-hour oxygen and index oxygenation index is the most valuable predictor of the prognosis of ARDS [22–24] .The selection of a 24-hour time point after aortic dissection surgery is in line with the internationally recognized characteristics of ARDS. Meanwhile, our study found that the oxygenation index was lower immediately and 24 h after aortic dissection surgery. At the same time, we found that 24-hour MS-ARDS significantly increased early mortality.Taking into account the above factors, we chose 24 h as the time point for judging MS-ARDS. In addition, the Berlin criteria indicate that the prognosis of moderate to severe ARDS is significantly worse than that of mild ARDS [15], and there are also other literature reports using moderate to severe ARDS as a predictive outcome indicator [25]. Therefore, we chose moderate to severe ARDS as our outcome.

At present, there is no corresponding prediction model for acute respiratory distress syndrome after STAAD. Therefore, we established the two prediction models through the machine learning method and compared their performance. XGboost is a model with high diagnostic efficiency rising in recent years and often appears in various machine learning competitions. Its full name is extreme gradient boosting. It is an advanced version of the decision tree model [21]. It was superior to the conventional decision tree model in finding the best prediction segmentation point and processing to improve efficiency [26]. Secondly, for the Logistic regression model, it can easily synthesize all types of variables as linear variables, which is more conducive to interpretation [27]. In our study, the impact of each variable on the MS-ARDS and the score of each variable can be clearly defined in Logistic regression. We compared the prediction efficiency of the two models. The logistic model has a higher positive prediction rate than XGboost. For patients at high risk of postoperative MS-ARDS, we can give priority to the logistic regression model to evaluate patients. However, the specificity of the XGboost model is higher than that of the Logistic model. We can use the XGboost model as an exclusion criterion. These two models have great clinical significance for the extraction time of endotracheal intubation after STAAD.

There are still some limitations to this research. First, for machine learning, our sample size is still small because of the low incidence of type A aortic dissection. Second, there were some missing data variables (more than 10%) in our research. We compared the different missing variables between the two groups, and there were significant differences in a small number of variables. We may have therefore missed some variables closely related to MS-ARDS and the missing samples may differ from the overall features, which may result in an imprecise description of the overall features. In addition, some variables may be related to other variables, such as operation time and cardiopulmonary bypass time. Finally, due to the retrospective nature of the study, there was a certain selection bias, and the baseline data of the two groups were different, which may have caused some deviation in the results.

## Conclusions

Postoperative MS-ARDS significantly increased early mortality after STAAD surgery. The LRM model has higher accuracy, and the XGBoost model has higher specificity.

### Supplementary Information

**Additional file 1: Supplemental Table 1.** Comparison of the missing variables between the MS-ARDS and non-MS-ARDS^a^. **Supplement Table 2.** Univariate logistic regression of all variables for postoperative MS-ARDS^a^. **Supplemental figure 1.** Forest map of factors affected MS-ARDS. **Supplemental figure 2. **Nomogram of categorical variables in logistic model **Supplement Table 3.** Univariate logistic regression of postoperative MS-ARDS^a^. **Supplemental figure 3. **A:ROC curve of stratified sampling logistic regression model B:ROC curve of stratified sampling XGboost model.

## Data Availability

The datasets used and/or analyzed during the current study are available from the corresponding author upon reasonable request.

## Abbreviations

TAAD: Type A aortic dissection
ARDS: Acute respiratory distress syndrome
MS-ARDS: moderate to severe acute respiratory distress syndrome
STAAD: Stanford type A aortic dissection. AUC,area under the receiver operating characteristic curve
LRM: Logistic regression model
BMI: Body mass index
COPD: Chronic obstructive pulmonary disease
CABG: Coronary artery bypass grafting

## References

[1] Zhao Y, Yue Y, Wang Y, Zhao W, Feng G (2021). The risk factors for postoperative acute respiratory distress syndrome in Stanford type a acute Aortic Dissection patients. Am J Translational Res.

[2] Liu N, Zhang W, Ma W, Shang W, Zheng J, Sun L (2017). Risk factors for hypoxemia following surgical repair of acute type a Aortic Dissection. Interact Cardiovasc Thorac Surg.

[3] Ketcham SW, Sedhai YR, Miller HC, Bolig TC, Ludwig A, Co I (2020). Causes and characteristics of death in patients with acute hypoxemic Respiratory Failure and acute respiratory distress syndrome: a retrospective cohort study. Crit Care (London England).

[4] Chen SW, Chang CH, Chu PH, Chen TH, Wu VC, Huang YK (2016). Risk factor analysis of postoperative acute respiratory distress syndrome in valvular heart Surgery. J Crit Care.

[5] Sinha P, Churpek MM, Calfee CS (2020). Machine learning classifier models can identify Acute respiratory distress syndrome phenotypes using readily available Clinical Data. Am J Respir Crit Care Med.

[6] Müller MC, Tuinman PR, Vlaar AP, Tuip AM, Maijoor K, Achouiti A (2014). Contribution of damage-associated molecular patterns to transfusion-related acute lung injury in cardiac Surgery. Blood Transfusion = Trasfusione del sangue.

[7] Morioka K, Muraoka R, Chiba Y, Ihaya A, Kimura T, Noguti H (1996). Leukocyte and platelet depletion with a blood cell separator: effects on lung injury after cardiac Surgery with cardiopulmonary bypass. J Thorac Cardiovasc Surg.

[8] Chen MF, Chen LW, Cao H, Lin Y (2016). Analysis of risk factors for and the prognosis of postoperative acute respiratory distress syndrome in patients with Stanford type A Aortic Dissection. J Thorac Disease.

[9] Fan E, Del Sorbo L, Goligher EC, Hodgson CL, Munshi L, Walkey AJ, An Official American Thoracic Society/European Society of Intensive Care Medicine/Society of Critical Care Medicine Clinical Practice Guideline (2017). Mechanical ventilation in adult patients with Acute Respiratory Distress Syndrome. Am J Respir Crit Care Med.

[10] Guérin C, Reignier J, Richard JC, Beuret P, Gacouin A, Boulain T (2013). Prone positioning in severe acute respiratory distress syndrome. N Engl J Med.

[11] Sanfilippo F, Palumbo GJ, Bignami E, Pavesi M, Ranucci M, Scolletta S (2022). Acute respiratory distress syndrome in the Perioperative period of cardiac Surgery: predictors, diagnosis, prognosis, Management options, and future directions. J Cardiothorac Vasc Anesth.

[12] Bein T, Weber-Carstens S, Goldmann A, Müller T, Staudinger T, Brederlau J (2013). Lower tidal volume strategy (≈ 3 ml/kg) combined with extracorporeal CO2 removal versus ‘conventional’ protective ventilation (6 ml/kg) in severe ARDS: the prospective randomized xtravent-study. Intensive Care Med.

[13] Terragni PP, Del Sorbo L, Mascia L, Urbino R, Martin EL, Birocco A (2009). Tidal volume lower than 6 ml/kg enhances lung protection: role of extracorporeal carbon dioxide removal. Anesthesiology.

[14] Sun L, Qi R, Zhu J, Liu Y, Zheng J (2011). Total arch replacement combined with stented elephant trunk implantation: a new standard therapy for type a dissection involving repair of the aortic arch?. Circulation.

[15] Ranieri VM, Rubenfeld GD, Thompson BT, Ferguson ND, Caldwell E, Fan E (2012). Acute respiratory distress syndrome: the Berlin definition. JAMA.

[16] Meduri GU, Headley S, Kohler G, Stentz F, Tolley E, Umberger R (1995). Persistent elevation of inflammatory cytokines predicts a poor outcome in ARDS. Plasma IL-1 beta and IL-6 levels are consistent and efficient predictors of outcome over time. Chest.

[17] Takir M, Kostek O, Ozkok A, Elcioglu OC, Bakan A, Erek A (2015). Lowering Uric Acid with Allopurinol improves insulin resistance and systemic inflammation in asymptomatic hyperuricemia. J Invest Medicine: Official Publication Am Federation Clin Res.

[18] Xie Q, Zhou Y, Xu Z, Yang Y, Kuang D, You H (2011). The ratio of CRP to prealbumin levels predict mortality in patients with hospital-acquired acute kidney injury. BMC Nephrol.

[19] Walker CM, Rosado-de-Christenson ML, Martínez-Jiménez S, Kunin JR, Wible BC (2015). Bronchial arteries: anatomy, function, hypertrophy, and anomalies. Radiographics: A Review Publication of the Radiological Society of North America Inc.

[20] Ahmed AH, Litell JM, Malinchoc M, Kashyap R, Schiller HJ, Pannu SR (2014). The role of potentially preventable hospital exposures in the development of acute respiratory distress syndrome: a population-based study. Crit Care Med.

[21] Smallwood CD, Boloori-Zadeh P, Silva MR, Gouldstone A (2017). High oxygen concentrations adversely affect the performance of pulmonary surfactant. Respir Care.

[22] Bernard GR, Artigas A, Brigham KL (1994). The american-european Consensus Conference on ARDS. Definitions, mechanisms, relevant outcomes, and clinical trial coordination. Am J Respir Crit Care Med.

[23] Artigas A, Carlet J, LeGall JR, Zapol L (1991). Clinical presentation, prognostic factors and outcome of ARDS in the European collaborative study. A preliminary report. Adult respiratory distress syndrome.

[24] Bone RC (1989). An early test of survival in patients with the adult respiratory distress syndrome: the Pao/FI02 ratio and its response to conventional therapy. Chest.

[25] Villar Jesús, González-Martín Jesús M, Hernández-González Jerónimo (2023). Predicting ICU mortality in Acute Respiratory Distress Syndrome patients using machine learning: the Predicting Outcome and STratifiCation of severity in ARDS (POSTCARDS) Study. Crit Care Med.

[26] Stapleton RD, Dixon AE, Parsons PE, Ware LB, Suratt BT (2010). The association between BMI and plasma cytokine levels in patients with acute lung injury. Chest.

[27] Xu HR, Yang Q, Xiang SY, Zhang PH, Ye Y, Chen Y (2021). Rosuvastatin enhances alveolar fluid clearance in Lipopolysaccharide-Induced Acute Lung Injury by activating the Expression of Sodium Channel and Na,K-ATPase via the PI3K/AKT/Nedd4-2 pathway. J Inflamm Res.

