# Supplementary material for: A scoring model based on clinical factors to predict postoperative moderate to severe acute respiratory distress syndrome in Stanford type A aortic dissection
Source: BMC Pulm Med. 2023 Dec 21;23:515. doi: 10.1186/s12890-023-02736-6 (PMC10734156; doi:10.1186/s12890-023-02736-6)
Supplement: Supplementary file 1 — Additional file 1: Supplemental Table 1. Comparison of the missing variables between the MS-ARDS and non-MS-ARDSa. Supplement Table 2. Univariate logistic regression of all variables for postoperative MS-ARDSa. Supplemental figure 1. Forest map of factors affected MS-ARDS. Supplemental figure 2. Nomogram of categorical variables in logistic model Supplement Table 3. Univariate logistic regression of postoperative MS-ARDSa. Supplemental figure 3. A:ROC curve of stratified sampling logistic regression model B:ROC curve of stratified sampling XGboost model. [file 12890_2023_2736_MOESM1_ESM.docx]

| Supplemental Table 1: Comparison of the missing variables between the MS-ARDS and non-MS-ARDS^a^ | | | | |
| --- | --- | --- | --- | --- |
| Variable | All patients | Non-MS-ARDS | MS-ARDS | *p*-*value* |
|  | n=594 | n=351 (59.1) | n=243 (40.9) |  |
| Troponin I, median (IQR) | 2.86 (5.38) | 2.405 (4.725) | 3.14 (5.97) | 0.206 |
| Creatine kinase-MB, median (IQR) | 15.8 (31.925) | 12.8 (32.05) | 17.2 (33.75) | 0.188 |
| Erythrocyte sedimentation rate, median (IQR) | 40.5 (43) | 40 (39) | 51 (83) | 0.409 |
| High-density lipoprotein, median (IQR) | 0.84 (0.56) | 0.86 (0.53) | 0.835 (0.69) | 0.674 |
| Low-density lipoprotein, median (IQR) | 2.37 (0.81) | 2.37 (0.88) | 2.26 (0.7975) | 0.583 |
| Serum total cholesterol, median (IQR) | 3.93 (1.37) | 3.93 (1.16) | 3.94 (1.4775) | 0.891 |
| Triglyceride, median (IQR) | 1.31 (0.885) | 1.33 (1.04) | 1.235 (0.93) | 0.979 |
| C-reactive protein, median (IQR) | 35.08 (12.95) | 33.78 (14.26) | 36.29 (12.675) | 0.045* |
| Base excess in interstitial fluid, median (IQR) | -2.2 (3.6) | -2.0 (3.8) | -2.6 (3.4) | 0.031* |
| Base excess, median (IQR) | -0.8 (3.1) | 7.485 (3.2) | -1 (3.025) | 0.056 |
| Diameter of aortic sinus, median (IQR) | 40 (9) | 42 (9) | 40 (8) | 0.053 |
| Ascending aorta diameter, median (IQR) | 45 (9) | 45 (9) | 45 (9) | 0.519 |
| Ejection fraction, median (IQR) | 61 (8) | 62 (8) | 60 (7) | 0.007* |
| Left ventricular end-diastolic dimension, median (IQR) | 50 (8) | 50 (9) | 49 (9) | 0.067 |
| Left ventricular end-systolic dimension, median (IQR) | 32 (7) | 32 (8) | 32 (7) | 0.899 |
| Ventricular septal thickness, median (IQR) | 12 (3) | 12 (3) | 12 (3) | 0.154 |
| Left ventricular posterior wall thickness, median (IQR) | 11 (2) | 11 (3) | 11 (2) | 0.044* |
| Left atrial diameter, median (IQR) | 35 (8) | 35 (8) | 36 (9) | 0.196 |
| Aortic stenosis, n (%) | 6 (1.0) | 2 (0.6) | 4 (1.6) | 0.216 |
| Aortic regurgitation, n (%) | 349 (58.8) | 211 (60.1) | 138 (56.8) | 0.82 |
| Mitral stenosis, n (%) | 1 (0.2) | 0 (0) | 1 (0.2) | 0.392 |
| Mitral insufficiency, n (%) | 146 (24.6) | 101 (28.8) | 45 (18.5) | 0.013* |
| Tricuspid stenosis, n (%) | 1 (0.2) | 0 (0) | 1 (0.2) | 0.392 |
| Tricuspid regurgitation, n (%) | 117 (19.7) | 86 (24.5) | 31 (12.8) | 0.001* |
| Aortic valve bilobar malformation, n (%) | 11 (1.9) | 7 (2.0) | 4 (1.6) | 1.000 |
| Pericardial effusion, n (%) | 81 (13.6) | 48 (13.7) | 33 (13.6) | 0.802 |
| Operation time, median (IQR) | 7.8 (2.1) | 7.5 (1.8) | 8.0 (2.3) | ＜0.001** |
| ASA anesthesia score, n (%) | 360 (60.6) | 204 (58.1) | 156 (64.2) | 0.925 |
| 1, n (%) | 2 (0.3) | 1 (0.3) | 1 (0.4) |  |
| 2, n (%) | 9 (1.5) | 6 (1.7) | 3 (1.2) |  |
| 3, n (%) | 158 (26.6) | 86 (24.5) | 72 (29.6) |  |
| 4, n (%) | 169 (28.5) | 98 (27.9) | 71 (29.2) |  |
| 5, n (%) | 22 (3.7) | 13 (3.7) | 9 (3.7) |  |
| The nasopharyngeal temperature during circulatory arrest, median (IQR) | 23.9 (1.7) | 23.8 (1.7) | 24.0 (1.6) | 0.326 |
| The anal temperature during circulatory arrest, median (IQR) | 25.3 (1.8) | 25.2 (2.1) | 25.4 (1.6) | 0.088 |
| a: Moderate to severe acute respiratory distress syndrome.**: p<0.01; *: p<0.05. | | | | |
|  |  |  |  |  |

| Supplement Table 2: Univariate logistic regression of all variables for postoperative MS-ARDS^a^ | | | |
| --- | --- | --- | --- |
| Variables | OR^b^ | 95%CI | *p-value* |
| Self-related factors |  |  |  |
| Age | 1.042 | 1.025-1.058 | ＜0.001** |
| Male | 0.814 | 0.563-1.177 | 0.275 |
| Temperature | 0.983 | 0.879-1.101 | 0.772 |
| Pulse | 1.013 | 1.001-1.025 | 0.033* |
| Systolic blood pressure | 1.008 | 1.000-1.017 | 0.061 |
| diastolic blood pressure | 1.017 | 1.005-1.029 | 0.004** |
| BMI^c^ | 1.164 | 1.109-1.223 | ＜0.001** |
| Onset to surgery | 0.958 | 0.895-1.024 | 0.206 |
| Hypertension | 1.869 | 1.335-2.617 | ＜0.001** |
| Cardiovascular history | 0.785 | 0.369-1.669 | 0.529 |
| Smoking | 1.162 | 0.825-1.635 | 0.391 |
| Diabetes | 2.227 | 0.897-5.533 | 0.085 |
| Valve surgery history | 2.905 | 0.262-32.212 | 0.385 |
| TEVAR history^d^ | 0.473 | 0.151-1.484 | 0.199 |
| PCI history^e^ | 1.448 | 0.203-10.351 | 0.712 |
| Storke history | 1.706 | 0.566-5.139 | 0.343 |
| Marfan history | 0.358 | 0.040-3.227 | 0.360 |
| coronary artery disease history | 2.937 | 0.727-11.858 | 0.130 |
| Cardiovascular factors |  |  |  |
| Diameter of aortic sinus | 0.978 | 0.954-1.003 | 0.086 |
| Ascending aorta diameter | 0.989 | 0.963-1.016 | 0.415 |
| Ejection fraction | 0.96 | 0.930-0.992 | 0.015* |
| Aortic stenosis | 4.724 | 0.488-45.799 | 0.180 |
| Ventricular septal thickness | 1.069 | 0.972-1.174 | 0.168 |
| Left ventricular posterior wall thickness | 1,111 | 1.002-1.233 | 0.046* |
| Left atrial diameter | 1.017 | 0.986-1.049 | 0.28 |
| Aortic insufficiency | 1.063 | 0.679-1.664 | 0.79 |
| Mitral stenosis | 1.554 | 0.097-25.000 | 0.756 |
| Mitral insufficiency | 0.586 | 0.386-0.890 | 0.012* |
| Tricuspid stenosis | 1.000 | 0 | 1.000 |
| Tricuspid insufficiency | 0.466 | 0.293-0.741 | 0.001** |
| Aortic valve bilobar malformation | 0.883 | 0.255-3.062 | 0.845 |
| Left ventricular end-diastolic diameter | 0.970 | 0.942-0.999 | 0.042* |
| Left ventricular end-systolic diameter | 0.998 | 0.962-1.037 | 0.932 |
| Inflammatory factors |  |  |  |
| Intraoperative blood transfusion | 1.077 | 1.019-1.139 | 0.009 |
| White blood cell | 1.026 | 0.987-1.066 | 0.197 |
| Mean corpuscular hemoglobin | 0.973 | 0.886-1.069 | 0.568 |
| Platelet | 0.995 | 0.992-0.998 | ＜0.001** |
| Red blood cell | 0.902 | 0.733-1.110 | 0.329 |
| Mean erythrocyte protein concentration | 0.992 | 0.977-1.007 | 0.309 |
| Hemoglobin | 0.997 | 0.990-1.004 | 0.391 |
| Neutrophils | 1.02 | 0.978-1.063 | 0.358 |
| Hematocrit | 1.005 | 0.972-1.038 | 0.785 |
| Mean red blood cell volume | 1.001 | 0.967-1.036 | 0.967 |
| Procalcitonin | 0.006 | 0.000-0.008 | ＜0.001** |
| Large platelet ratio | 1.009 | 0.987-1.031 | 0.422 |
| Percentage of neutrophils | 0.999 | 0.989-1.010 | 0.906 |
| Blood urea nitrogen | 1.126 | 1.076-1.179 | ＜0.001** |
| Creatinine | 1.011 | 1.007-1.014 | ＜0.001** |
| Uric acid | 1.005 | 1.003-1.006 | ＜0.001** |
| Alanine aminotransferase | 1.001 | 1.000-1.002 | 0.08 |
| Aspartate aminotransferase | 1.001 | 1.000-1.001 | 0.049* |
| Alkaline phosphatase | 1.001 | 0.996-1.006 | 0.745 |
| Serum total bile acid | 1.106 | 0.996-1.228 | 0.06 |
| Plasma prothrombin time | 0.991 | 0.984-0.998 | 0.016* |
| Red blood cell distribution width | 1.166 | 1.037-1.311 | 0.01* |
| Serum indirect bilirubin | 1.006 | 1.000-1.011 | 0.062 |
| Serum direct bilirubin | 1.011 | 1.002-1.021 | 0.018* |
| Albumin to globulin ratio | 0.864 | 0.617-1.208 | 0.392 |
| Glutamine transferase | 1 | 0.997-1.003 | 0.894 |
| Serum cholinesterase | 0.941 | 0.844-1.048 | 0.268 |
| Serum fibrinogen | 0.966 | 0.845-1.105 | 0.615 |
| Fibrin degradation products | 1.002 | 0.997-1.008 | 0.363 |
| D-Dimer | 1 | 1.000-1.000 | 0.279 |
| International normalized ratio | 1.032 | 0.969-1.098 | 0.329 |
| Surgical factors |  |  |  |
| Sun's classification(Type 2 vs Type 1) | 0.664 | 0.436-1.010 | 0.056 |
| Sun's classification(Type 3 vs Type 1) | 0.356 | 0.220-0.578 | ＜0.001** |
| Sun's classification(Complex vs simple) | 1.494 | 0.633-3.522 | 0.359 |
| Cardiopulmonary bypass time | 1.009 | 1.005-1.013 | ＜0.001** |
| Aortic occlusion time | 1.006 | 1.001-1.010 | 0.015* |
| Operation time | 1.195 | 1.072-1.332 | 0.001** |
| Cardiac Arrest time | 1.028 | 1.010-1.047 | 0.003** |
| Concomitant CABG^f^ | 2.456 | 1.205-5.008 | 0.013* |
| Brachiocephalic vascular bypass | 1.399 | 0.819-2.389 | 0.219 |
| Nasopharyngeal temperature | 0.993 | 0.886-1.114 | 0.909 |
| Anal temperature | 1.075 | 0.965-1.197 | 0.190 |
| Proximal surgery |  |  |  |
| Ascending aorta replacement | 1.148 | 0.741-1.778 | 0.537 |
| Bentall | 0.506 | 0.346-0.740 | ＜0.001** |
| Wheat | 1.13 | 0.070-18.303 | 0.931 |
| David | 0 | 0 | 1.000 |
| Aortic arch surgery |  |  |  |
| Total arch replacement | 1.288 | 0.858-1.931 | 0.222 |
| Half arch replacement | 1.35 | 0.603-3.023 | 0.465 |
| pulmonary edema |  |  |  |
| Total plasma protein | 0.97 | 0.952-0.988 | 0.001** |
| Albumin | 0.945 | 0.916-0.974 | ＜0.001** |
| Prealbumin | 0.004 | 0.000-0.190 | 0.005** |
| Preoperative lung injury factors |  |  |  |
| PCO2 | 0.956 | 0.924-0.989 | 0.009** |
| Base excess in interstitial fluid | 0.947 | 0.894-1.003 | 0.064 |
| Base excess | 0.951 | 0.890-1.015 | 0.132 |
| Standard base excess | 0.938 | 0.869-1.013 | 0.103 |
| PO2 | 0.998 | 0.997-1.000 | 0.098 |
| Preoperative aspiration |  |  |  |
| Pain | 1.243 | 0.846-1.827 | 0.267 |
| Limb numbness | 1.09 | 0.578-2.055 | 0.790 |
| nausea | 1.32 | 0.790-2.207 | 0.289 |
| Sweating | 1.225 | 0.806-1.862 | 0.342 |
| Palpitation | 2.905 | 0.262-32.212 | 0.385 |
| Dizzy | 1.9 | 0.946-3.818 | 0.710 |
| headache | 1.464 | 0.600-3.572 | 0.403 |
| syncope | 2.921 | 0.531-16.072 | 0.218 |
| Chest tightness | 0.915 | 0.527-1.587 | 0.751 |
| suffocation | 0.805 | 0.350-1.853 | 0.611 |
| Shortness of breath | 2.053 | 0.644-6.544 | 0.224 |
| Chest pain | 0.773 | 0.529-1.129 | 0.183 |
| Chest and back pain | 1.383 | 0.994-1.924 | 0.054 |
| Back pain | 1.673 | 0.599-4.676 | 0.326 |
| Limb pain | 1.752 | 0.529-5.806 | 0.359 |
| Vomit | 1.454 | 0.855-2.473 | 0.167 |
| a: Moderate to severe acute respiratory distress. b: Odd ratio.c: body mass index. d: Thoracic endovascular aortic repair.e: Percutaneous coronary intervention.f: Coronary artery bypass grafting. | | | |

| Supplement Table 3: Univariate logistic regression of postoperative MS-ARDSa | | | |
| --- | --- | --- | --- |
| Variables | OR^b^ | 95%CI | *p-value* |
| Self-related factors |  |  |  |
| Age | 1.042 | 1.025-1.059 | ＜0.001** |
| Male | 0.814 | 0.563-1.177 | 0.275 |
| Temperature | 0.983 | 0.879-1.101 | 0.772 |
| Pulse | 1.013 | 1.001-1.025 | 0.033* |
| Systolic blood pressure | 1.008 | 1.000-1.017 | 0.061 |
| diastolic blood pressure | 1.017 | 1.005-1.029 | 0.004** |
| BMI^c^ | 1.164 | 1.109-1.223 | ＜0.001** |
| Onset to surgery | 0.958 | 0.895-1.024 | 0.206 |
| Hypertension | 1.869 | 1.335-2.617 | ＜0.001** |
| Cardiovascular history | 0.785 | 0.369-1.669 | 0.529 |
| Smoking | 1.162 | 0.825-1.635 | 0.391 |
| Diabetes | 2.227 | 0.897-5.533 | 0.085 |
| Valve surgery history | 2.905 | 0.262-32.212 | 0.385 |
| TEVAR history^d^ | 0.473 | 0.151-1.484 | 0.199 |
| PCI history^e^ | 1.448 | 0.203-10.351 | 0.712 |
| Storke history | 1.706 | 0.566-5.139 | 0.343 |
| Marfan history | 0.358 | 0.040-3.227 | 0.360 |
| coronary artery disease history | 2.937 | 0.727-11.858 | 0.130 |
| Inflammatory factors |  |  |  |
| White blood cell | 1.026 | 0.987-1.066 | 0.197 |
| Mean erythrocyte hemoglobin | 0.973 | 0.886-1.069 | 0.568 |
| Platelet | 0.995 | 0.992-0.998 | ＜0.001** |
| Red blood cell | 0.902 | 0.733-1.110 | 0.329 |
| Mean erythrocyte protein concentration | 0.992 | 0.977-1.007 | 0.309 |
| Hemoglobin | 0.997 | 0.990-1.004 | 0.391 |
| Neutrophils | 1.02 | 0.978-1.063 | 0.358 |
| Hematocrit | 1.005 | 0.972-1.038 | 0.785 |
| Mean corpuscular volume | 1.001 | 0.967-1.036 | 0.967 |
| Procalcitonin | 0.006 | 0.000-0.008 | ＜0.001** |
| Large platelet ratio | 1.009 | 0.987-1.031 | 0.422 |
| Red blood cell distribution width | 1.166 | 1.037-1.311 | 0.01* |
| Percentage of neutrophils | 0.999 | 0.989-1.010 | 0.906 |
| Blood urea nitrogen | 1.126 | 1.076-1.179 | ＜0.001** |
| Creatinine | 1.011 | 1.007-1.014 | ＜0.001** |
| Uric acid | 1.005 | 1.003-1.006 | ＜0.001** |
| Alanine aminotransferase | 1.001 | 1.000-1.002 | 0.08 |
| Aspartate aminotransferase | 1.001 | 1.000-1.001 | 0.049* |
| Alkaline phosphatase | 1.001 | 0.996-1.006 | 0.745 |
| Total bile acid | 1.106 | 0.996-1.228 | 0.06 |
| Prothrombin time | 0.991 | 0.984-0.998 | 0.016* |
| Total bilirubin | 1.006 | 1.000-1.011 | 0.062 |
| Direct bilirubin | 1.011 | 1.002-1.021 | 0.018* |
| Albumin to globulin ratio | 0.864 | 0.617-1.208 | 0.392 |
| Glutamine transferase | 1 | 0.997-1.003 | 0.894 |
| Serum cholinesterase | 0.941 | 0.844-1.048 | 0.268 |
| Fibrinogen | 0.966 | 0.845-1.105 | 0.615 |
| Fibrinogen degradation products | 1.002 | 0.997-1.008 | 0.363 |
| D-Dimer | 1 | 1.000-1.000 | 0.279 |
| International normalized ratio | 1.032 | 0.969-1.098 | 0.329 |
| Surgical factors |  |  |  |
| Sun's classification(Type 2 vs Type 1) | 0.664 | 0.436-1.010 | 0.056 |
| Sun's classification(Type 3 vs Type 1) | 0.356 | 0.220-0.578 | ＜0.001** |
| Sun's classification(Complex vs simple) | 1.494 | 0.633-3.522 | 0.359 |
| Cardiopulmonary bypass time | 1.009 | 1.005-1.013 | ＜0.001** |
| Aortic occlusion time | 1.006 | 1.001-1.010 | 0.015* |
| Operation time | 1.195 | 1.072-1.332 | 0.001** |
| Cardiac Arrest time | 1.028 | 1.010-1.047 | 0.003** |
| Concomitant CABG^f^ | 2.456 | 1.205-5.008 | 0.013* |
| Brachiocephalic vascular bypass | 1.399 | 0.819-2.389 | 0.219 |
| Nasopharyngeal temperature | 0.993 | 0.886-1.114 | 0.909 |
| Anal temperature | 1.075 | 0.965-1.197 | 0.190 |
| Proximal surgery |  |  |  |
| Ascending aorta replacement | 1.148 | 0.741-1.778 | 0.537 |
| Bentall | 0.506 | 0.346-0.740 | ＜0.001** |
| Wheat | 1.13 | 0.070-18.303 | 0.931 |
| David | 0 | 0 | 1.000 |
| Aortic arch surgery |  |  |  |
| Total arch replacement | 1.288 | 0.858-1.931 | 0.222 |
| Half arch replacement | 1.350 | 0.603-3.023 | 0.465 |
| pulmonary edema |  |  |  |
| Total protein | 0.970 | 0.952-0.988 | 0.001** |
| Albumin | 0.945 | 0.916-0.974 | ＜0.001** |
| Prealbumin | 0.004 | 0.000-0.190 | 0.005** |
| Preoperative lung injury factors |  |  |  |
| PCO2 | 0.956 | 0.924-0.989 | 0.009** |
| Base excess in interstitial fluid | 0.947 | 0.894-1.003 | 0.064 |
| Base excess | 0.951 | 0.890-1.015 | 0.132 |
| Standard base excess | 0.938 | 0.869-1.013 | 0.103 |
| PO2 | 0.998 | 0.997-1.000 | 0.098 |
| Preoperative aspiration |  |  |  |
| Pain | 1.243 | 0.846-1.827 | 0.267 |
| Limb numbness | 1.090 | 0.578-2.055 | 0.790 |
| nausea | 1.320 | 0.790-2.207 | 0.289 |
| Sweating | 1.225 | 0.806-1.862 | 0.342 |
| Palpitation | 2.905 | 0.262-32.212 | 0.385 |
| Dizzy | 1.900 | 0.946-3.818 | 0.710 |
| headache | 1.464 | 0.600-3.572 | 0.403 |
| syncope | 2.921 | 0.531-16.072 | 0.218 |
| Chest tightness | 0.915 | 0.527-1.587 | 0.751 |
| suffocation | 0.805 | 0.350-1.853 | 0.611 |
| Shortness of breath | 2.053 | 0.644-6.544 | 0.224 |
| Chest pain | 0.773 | 0.529-1.129 | 0.183 |
| Chest and back pain | 1.383 | 0.994-1.924 | 0.054 |
| Back pain | 1.673 | 0.599-4.676 | 0.326 |
| Limb pain | 1.752 | 0.529-5.806 | 0.359 |
| Vomit | 1.454 | 0.855-2.473 | 0.167 |
| a: Moderate to severe acute respiratory distress. b: Odd ratio.c: body mass index. d: Thoracic endovascular aortic repair.e: Percutaneous coronary intervention.f: Coronary artery bypass grafting. | | | |


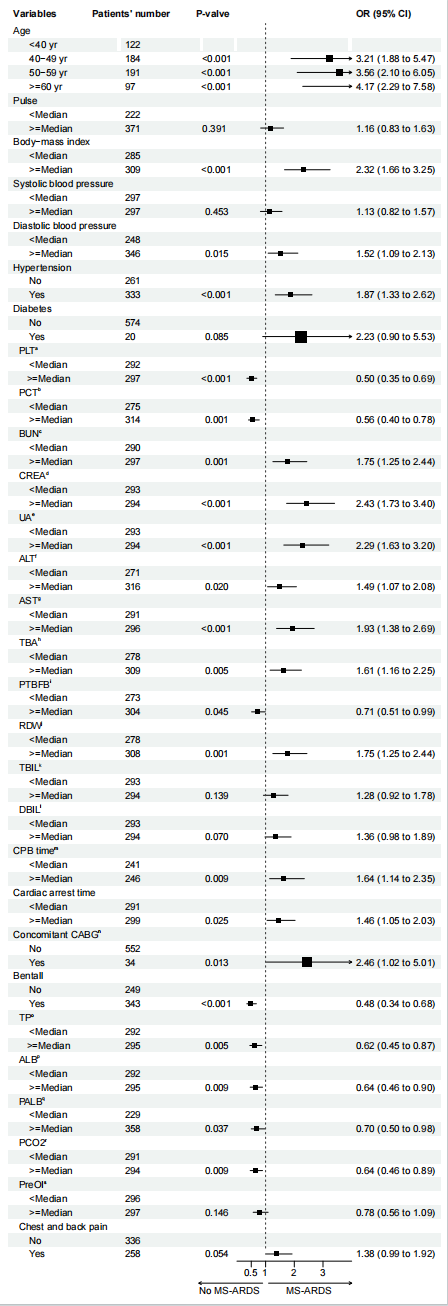


**Supplemental figure 1:**forest map of factors affected MS-ARDS

(a:Platelet; b: Procalcitonin; c: Blood urine nitrogen; d: Creatinine; e: Uric acid; f: Alanine aminotransferase; g: Aspartate transaminase; h: Total bile acid; i: Plasma prothrombin time; j: Red blood cell distribution width; k: Total bilirubin; l: Direct bilirubin; m: Cardiopulmonary bypass time; n: Concomitant coronary bypass grafting surgery; o: Total plasma protein; p: Albumin; q: Prealbumin; r: Carbon dioxide partial pressure; s: Preoperative oxygenation inde; OR: odds ratio; 95CI: 95% confidence interval .)

**
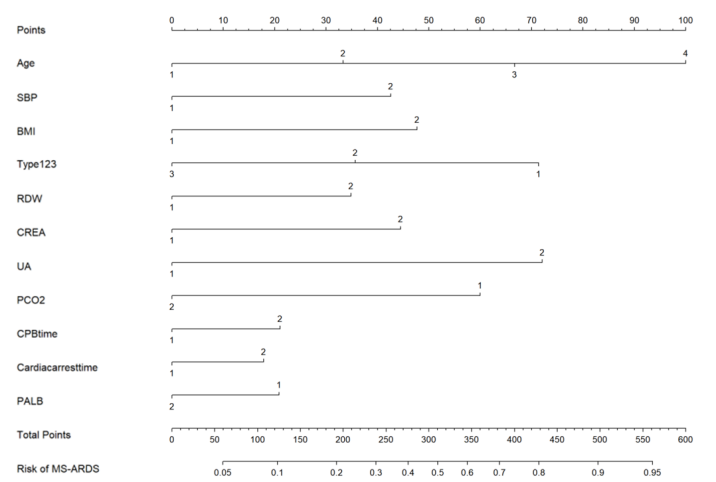
**

**Supplemental figure 2：**Nomogram of categorical variables in logistic model

(SBP: systolic blood pressure; BMI: body mass index; Type123: Sun’s classification 1,2,3; RDW: Red blood cell distribution width; CREA: Creatinine; UA:Uric acid; PCO2:Preoperative Carbon dioxide partial pressure; CPBtime:cardiopulmonary bypass time; PALB:Prealbumin)


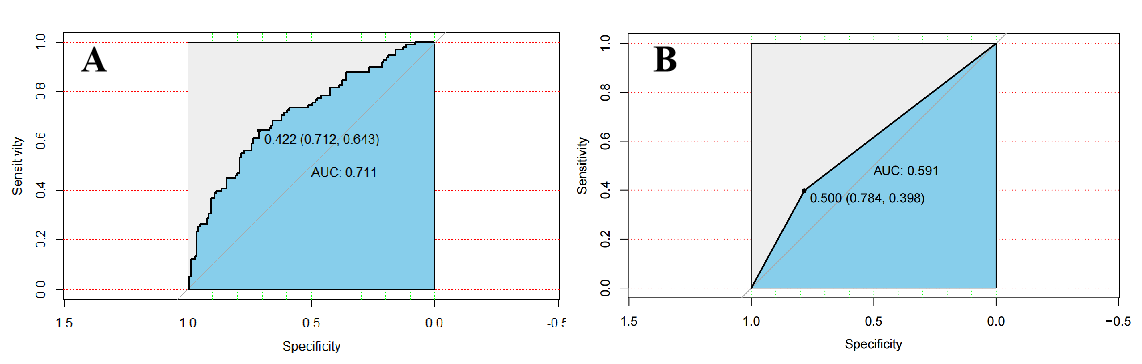


**Supplemental figure 3:**A:ROC curve of stratified sampling logistic regression model

B:ROC curve of stratified sampling XGboost model
